# Gender Differences in Electrophysiological Gene Expression in Failing and Non-Failing Human Hearts

**DOI:** 10.1371/journal.pone.0054635

**Published:** 2013-01-23

**Authors:** Christina M. Ambrosi, Kathryn A. Yamada, Jeanne M. Nerbonne, Igor R. Efimov

**Affiliations:** 1 Department of Biomedical Engineering, Washington University in St. Louis, St. Louis, Missouri, United States of America; 2 Department of Medicine, Washington University School of Medicine, St. Louis, Missouri, United States of America; 3 Department of Developmental Biology, Washington University School of Medicine, St. Louis, Missouri, United States of America; University of Minnesota, United States of America

## Abstract

The increasing availability of human cardiac tissues for study are critically important in increasing our understanding of the impact of gender, age, and other parameters, such as medications and cardiac disease, on arrhythmia susceptibility. In this study, we aimed to compare the mRNA expression of 89 ion channel subunits, calcium handling proteins, and transcription factors important in cardiac conduction and arrhythmogenesis in the left atria (LA) and ventricles (LV) of failing and nonfailing human hearts of both genders. Total RNA samples, prepared from failing male (n = 9) and female (n = 7), and from nonfailing male (n = 9) and female (n = 9) hearts, were probed using custom-designed Taqman gene arrays. Analyses were performed to explore the relationships between gender, failure state, and chamber expression. Hierarchical cluster analysis revealed chamber specific expression patterns, but failed to identify disease- or gender-dependent clustering. Gender-specific analysis showed lower expression levels in transcripts encoding for K_v_4.3, KChIP2, K_v_1.5, and K_ir_3.1 in the failing female as compared with the male LA. Analysis of LV transcripts, however, did not reveal significant differences based on gender. Overall, our data highlight the differential expression and transcriptional remodeling of ion channel subunits in the human heart as a function of gender and cardiac disease. Furthermore, the availability of such data sets will allow for the development of disease-, gender-, and, most importantly, patient-specific cardiac models, with the ability to utilize such information as mRNA expression to predict cardiac phenotype.

## Introduction

Epidemiologically, there are well-established gender differences in the manifestations of cardiac arrhythmias. Men are more susceptible to the development of atrial fibrillation (AF) [1], [2], whereas women have higher incidence of long-QT syndrome (LQTS) and drug-induced Torsades de Pointes [3], [4]. In addition, men and women exhibit differences in basic electrophysiological parameters during normal sinus rhythm. Women, for example, tend to have increased resting heart rates [5] and longer rate-corrected QT (QTc) intervals [6], whereas men have been reported to have longer P-waves suggesting differences in baseline atrial electrophysiological properties [7]. Interestingly, autonomic blockade does not mask the gender differences in resting heart rate, suggesting that intrinsic sinus node differences, as opposed to gender dependent autonomic tone, are responsible for this variability [5].

Much less is known, however, about the molecular mechanisms underlying observed gender differences in arrhythmia susceptibility and basic electrophysiological parameters in the human heart. Insight has been gained primarily from animal models, particularly studies in the rabbit, dog, and mouse [8]. The rabbit heart has been suggested to be a relevant model for the study of human arrhythmias [9] and female rabbits also exhibit gender dependent differences in QTc intervals [10] similar to that seen in humans [6]. Although mice afford easy genetic manipulations, they manifest profound differences in cardiac electrophysiology compared to humans [11], [12] which must be considered when extending conclusions to human physiology and pathophysiology [13], [14]. Since the expression profile of ion channels and transporters determine cardiac excitability and may also determine arrhythmia susceptibility, several recent studies have explored gene expression patterns in the human heart [15], [16], [17], [18], [19], [20]. As the ever present limitation to detailed human studies is the availability of human tissues for analysis, we have developed the infrastructure necessary to collect and systematically investigate tissues from both failing and nonfailing human hearts suitable for detailed functional and molecular analyses. In addition to investigating the global patterns of mRNA expression relating to gender and heart failure in the human heart, we have also provided a comprehensive set of data which was used to computationally model patient-specific electrophysiology and explore population-dependent variability in action potential morphology by our colleagues Walmsley, et al.

We focused on probing tissues of the left atria (LA) and ventricle (LV), as left-sided heart remodeling and dysfunction are the most common manifestations of heart failure [21], [22]. Specifically, we quantified the transcript expression levels of 89 target genes in the LA and LV of failing and nonfailing human hearts of both genders. In some analyses, tissues isolated from the epicardium and endocardium of the LV were examined separately as we have previously shown that heart failure results in a heterogeneous remodeling of repolarization across the ventricular wall and altered dispersion [23], which may be pro-arrhythmic [24].

## Methods

The use of human hearts for research was approved by the Institutional Review Board at Washington University in St. Louis.

### Human Tissue Samples

**Figure 1:**
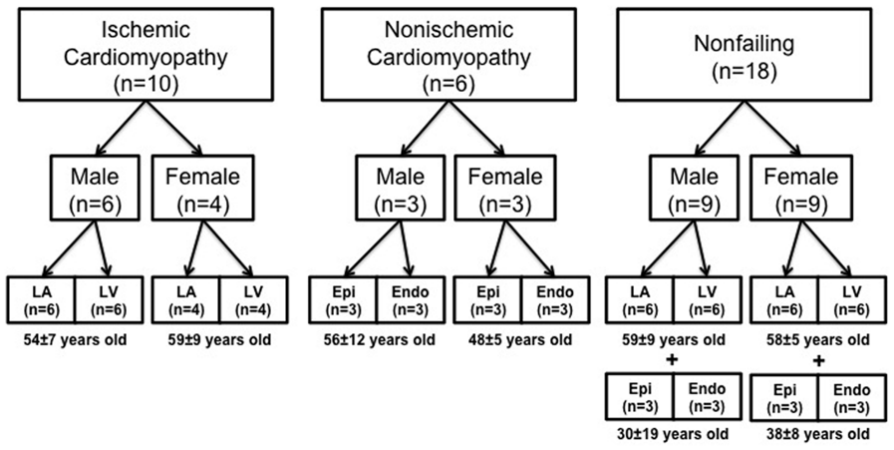
Study population (n = 34).

For this study a total of 34 explanted human hearts of both genders (n = 16 women, 52±10 years old; n = 18 men, 52±14 years old) were acquired from two sources (Figure 1). Failing human hearts (n = 16; 9 men, 7 women) were obtained at the time of cardiac transplantation at Barnes-Jewish Hospital (St. Louis, MO) and nonfailing hearts (n = 18; 9 men, 9 women) were provided by Mid-America Transplant Services (St. Louis, MO) after they were deemed unsuitable for cardiac transplantation. Each heart was collected in the operating room after removal from the chest and immediately perfused with and stored in cardioplegic solution (4°C) for preservation during the 15–20 minute delivery from the operating room to the research laboratory. Upon arrival, tissue samples from the LA (n = 22) and LV (n = 22) free walls were immediately harvested and preserved. Epicardial and endocardial samples were also harvested from the LV free wall (n = 12). All tissue samples were preserved and stored in RNAlater (Sigma-Aldrich, St. Louis, MO) at −80°C. Basic clinical characteristics of the patients are shown in Tables 1 and 2.

**Table 1:** Clinical characteristics of nonfailing hearts.

HeartNumber	Gender	Age	Cause of Death	LVEDD* (cm)	EF† (%)
1	Female	55	Stroke	unknown	<20
2	Female	33	Head Trauma	unknown	unknown
3	Female	59	Anoxic Brain Injury	unknown	unknown
4	Female	66	Stroke	unknown	unknown
5	Female	34	Brain Death	4.8	20
6	Female	58	Stroke	unknown	30
7	Female	49	Anoxia	4.1	65
8	Female	59	unknown	unknown	unknown
9	Female	50	Anoxia	unknown	60–65
10	Male	19	Anoxia	4.1	45
11	Male	60	Head Trauma	3.3	65
12	Male	55	Stroke	4.7	35
13	Male	50	Stroke	unknown	unknown
14	Male	52	Head Trauma	4.3	60–70
15	Male	65	Stroke	unknown	unknown
16	Male	58	Stroke	unknown	60–70
17	Male	20	Drug Overdose	unknown	unknown
18	Male	59	Brain Death	unknown	unknown

* left ventricular end diastolic dimension.

† ejection fraction.

**Table 2:** Clinical characteristics of failing hearts.

Heart Number	Gender	Age	Diagnosis	Devices	LVEDD* (cm)	EF† (%)	Arrhythmia History
1	Female	65	ICM‡	ICD§	6.4	15	NSVT||, VF#
2	Female	49	ICM	ICD	5.9	25–30	VT**
3	Female	53	ICM	ICD	6.5	20	No arrhythmias
4	Female	67	ICM	ICD	7	10	No arrhythmias
5	Male	50	ICM	PPM^a^, ICD, BiV^b^	7	<15	Refractory VT
6	Male	44	ICM	ICD, LVAD^c^	7	<15	No arrhythmias
7	Male	69	ICM	PPM, ICD, BiV	7.13	39	AF^d^, VT
8	Male	63	ICM	PPM, ICD, BiV	7.6	15	No arrhythmias
9	Male	50	ICM	ICD	6.6	30	NSVT, VF
10	Male	67	ICM	ICD, LVAD	7.9	10	No arrhythmias
11	Female	44	NICM^e^	ICD, LVAD	5.5	20–25	AF
12	Female	54	NICM	ICD	6.1	<20	AF, VT
13	Female	46	NICM	ICD, LVAD	7.8	16	AF
14	Male	70	NICM	ICD	>6.6	16	AF
15	Male	47	NICM	LVAD	6.4	25	VT
16	Male	53	NICM	ICD	8.2	16	VT

* left ventricular end diastolic volume

** ventricular tachycardia.

† ejection fraction

a permanent pacemaker.

‡ ischemic cardiomyopathy

b biventricular device.

§ implantable cardioverter defibrillator

c left ventricular assist device.

|| nonsustained ventricular tachycardia

d atrial fibrillation.

# ventricular fibrillation

e nonischemic cardiomyopathy.

### RNA Extraction and Preparation

Total RNA was extracted from 68 samples using the RNEasy Fibrous Tissue Mini Kit (Qiagen, Valencia, CA) according to the manufacturer’s instructions. RNA yield was measured using a Nanodrop 1000 (Thermo Scientific) and quality was assessed by verifying the presence of the 18 S and 28 S bands as run on a 1% agarose gel.

### Low-Density Taqman Gene Arrays

Custom-designed low-density Taqman gene arrays (Applied Biosytems, Foster City, CA) were used to probe for the presence of 96 targets in a two-step process as described previously [16], [19], [25]. In short, total RNA (1–2 µg) was first converted to cDNA using the High Capacity cDNA Reverse Transcription Kit (Applied Biosystems, Foster City, CA). Subsequent reactions were performed, using 100 ng of cDNA loaded into each well of our custom-designed gene arrays, with the ABI PRISM 7900 HT Sequence Detection System (Applied Biosystems, Foster City, CA). Genes selected for analysis included 56 ion channels and accessory proteins, 14 calcium-handling proteins, 9 autonomic receptors, 6 transcription factors, 3 signaling proteins, and 8 controls/marker genes and are listed in Table S1.

### Statistics and Data Analysis

Data were collected and analyzed by the Applied Biosystems SDS 2.3 software using the threshold cycle (C_t_) relative quantification method [26] with GAPDH as an endogenous control. Quantitative data are expressed as mean ± standard deviation. Statistically significant differences were identified using a one-way analysis of variance followed by Tukey-Kramer’s test with a significance level of p<5.6×10^−4^ based on the Bonferroni correction (GenEx; MultiD, Santa Clara, CA). All quantitative results are available at the NIH GEO database (GEO Accession #GSE33165).

In addition, two-way hierarchical agglomerative cluster analysis was performed using the ΔΔC_t_ of each target. We applied average linkage clustering with calculation of Euclidean distances. Clusters were analyzed and visualized using Cluster 3.0 and Treeview software [27].

## Results

### Hierarchical Cluster Analysis

Hierarchical cluster analysis was used to determine if strong enough overall trends in transcript expression across gender, disease, and cardiac chambers manifested to create distinct clusters of highly-related samples. As is shown in the Online Data Supplement (Figure S1), LA and LV samples from ischemic and nonfailing hearts of both genders show distinct clustering based on chamber location (Figure S1A). These samples do not cluster, however, based on gender or disease state. We also explored cluster analysis of ischemic, nonischemic, and nonfailing LV samples of both genders (Figure S1B), as well as nonischemic and nonfailing samples from the epicardium and endocardium of the LV (Figure S1C). Clustering of these latter samples based on gender, disease, or cardiac location was not evident.

### Ventricular Remodeling in Heart Failure and Differences in Transcript Expression

**Figure 2:**
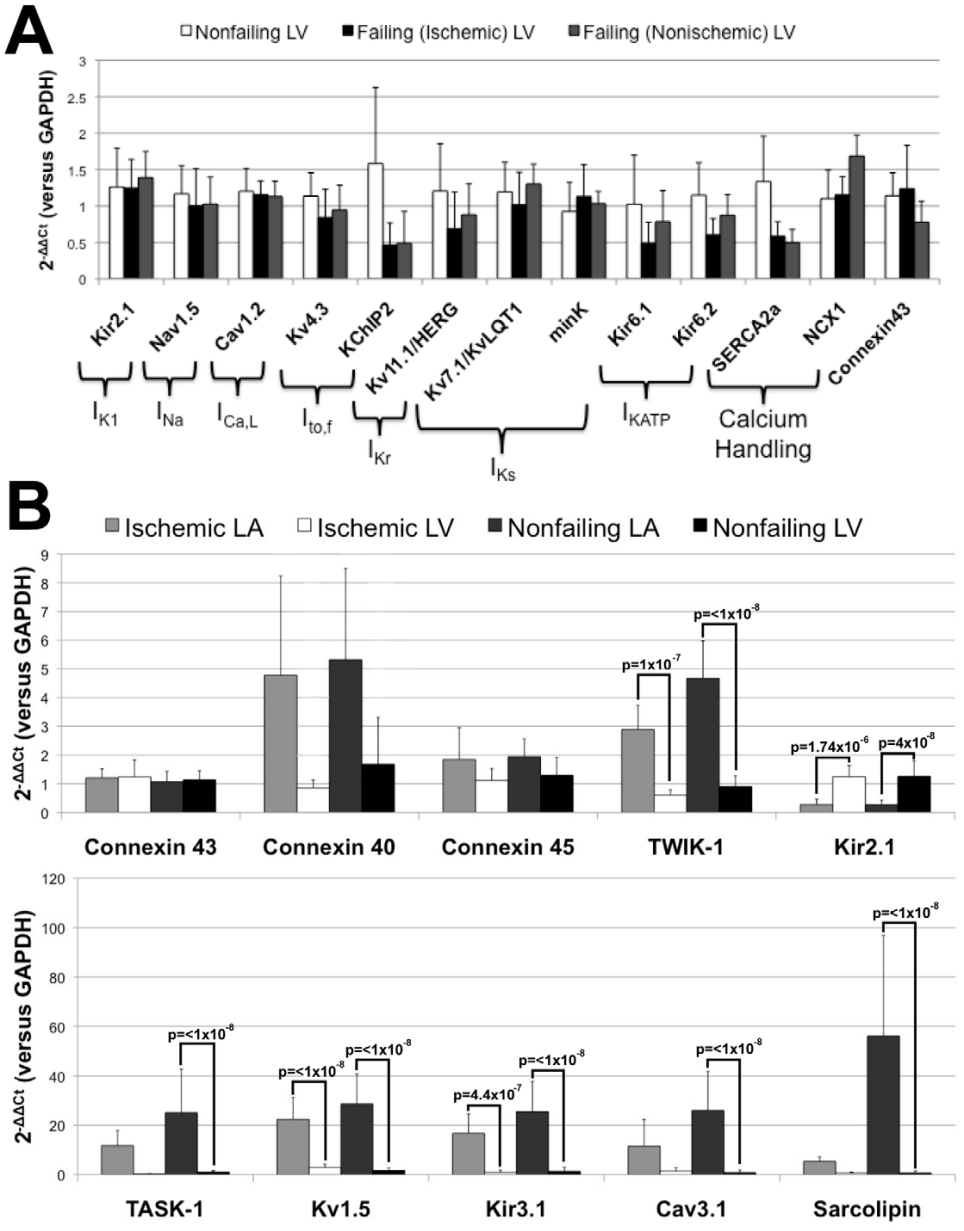
Ventricular remodeling in heart failure. (A) Ventricular remodeling of major ion channel subunits and accessory proteins in heart failure. (B) Regional specificity of targets in failing and nonfailing hearts of both genders.

Previous studies have revealed that during the heart failure remodeling process, the cardiac action potential is significantly prolonged and conduction is slowed due to pathological changes in the expression and function of ion channels [28], [29]. As shown in Figure 2A, we observe trends for reduction in the expression of transcripts encoding ion channel subunits that are important in cardiac conduction and repolarization, as well as changes in key calcium handling proteins. Similar to previously reported findings, for example, we observe downregulation of the subunits encoding for I_KATP_ [30] and I_to_ [31]. I_to,fast_ channels, in particular, encoded by the pore-forming subunit K_v_4.3 and the accessory protein KChIP2, is one of the most consistently altered ion channels during the heart failure remodeling process across species [32]. Consistent with previous suggestions, reductions in I_to,fast_ density in the failing heart are linked to protein-level downregulation of K_v_4.3 and transcript-level downregulation of KChIP2 [33]. We find lower KChIP2 expression in LV samples from failing, compared with nonfailing hearts (Figure 2A). In contrast with the findings for KChIP2, we observe no significant changes in the expression of subunits encoding I_K1_, I_Na_, and I_Ca,L_ channels. There is, however, a trend for lower expression of K_v_11.1/HERG, the subunit encoding the rapid component of delayed rectification, I_Kr_, as well as the calcium handling proteins, SERCA2a and NCX1, consistent with recent findings [33]. Finally, we observe a downregulation of connexin 43 in nonischemic, but not ischemic, cardiomyopathy.

Major differences in atrial-ventricular transcript expression patterns are shown in Figure 2B. Consistent with previous findings, we observed atrial predominance of several transcripts, such as connexin 40, TASK-1, TWIK-1, K_ir_2.1, K_v_1.5, K_ir_3.1, Ca_v_3.1, and sarcolipin [18], [34], [35]. In addition, with the exception of connexin 40, we measured similar expression levels of the connexin 40, 45, and 43 transcript in both the LA and LV (Figure 2B).

The general agreement of our findings, regarding both ventricular remodeling during heart failure and atrio-ventricular specificity, with previous studies [18], [30], [31], [34], [35] both supports the validity of our findings and allows for the exploration of other novel gene expression patterns with regard to gender and arrhythmia susceptibility.

### Gender Dependent Atrial/Ventricular Bias

**Figure 3:**
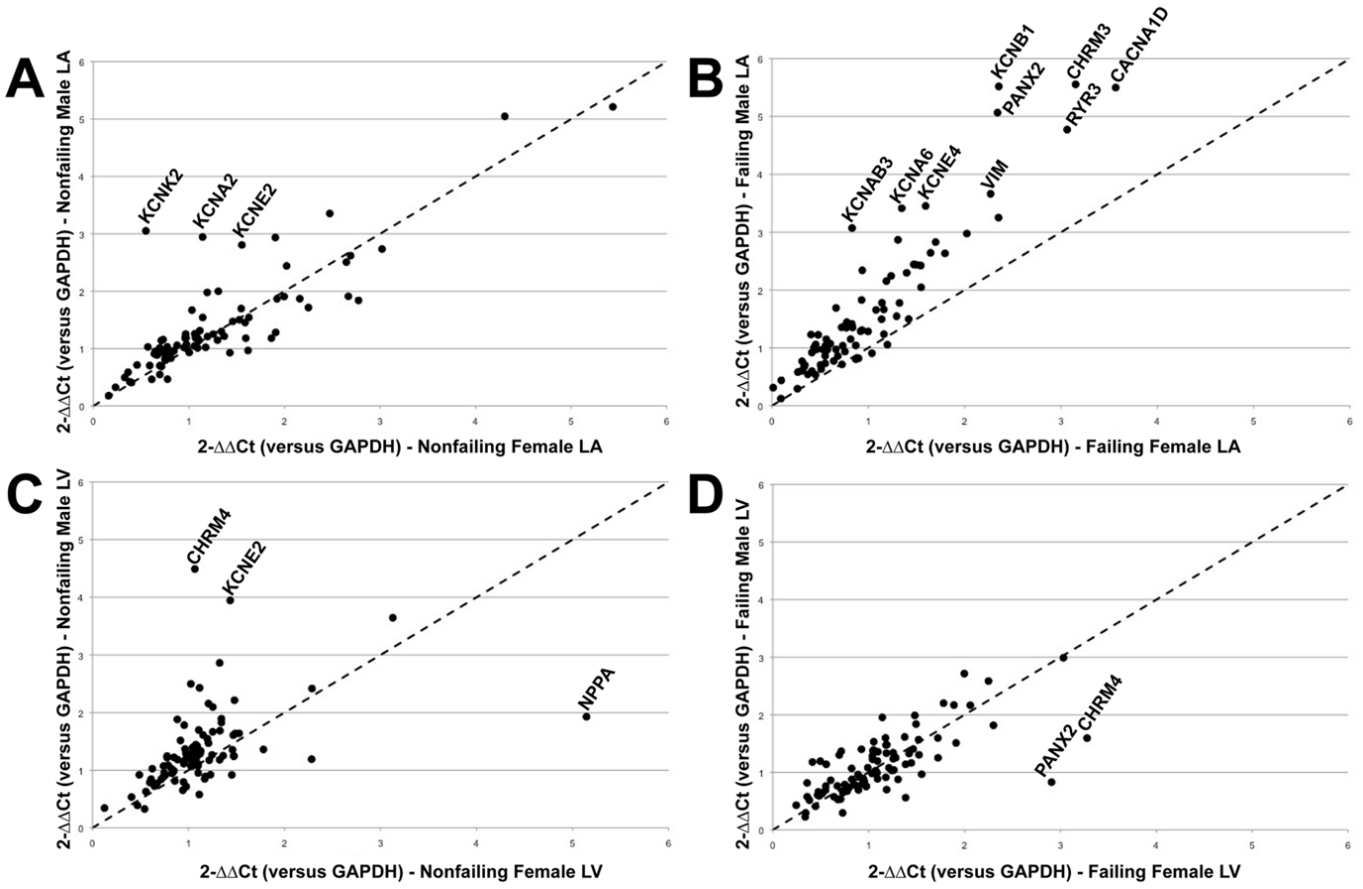
Gender dependent comparison of relative expression levels of all genes. (A) Nonfailing male versus female LA. (B) Failing male versus female LA showing a distinct male bias. (C) Nonfailing male versus female LV. (D) Failing female versus male LV. The dotted diagonal line represents equal expression levels between genders.

A gender-based comparison of overall relative expression levels for all targets in both failing and nonfailing hearts is shown in Figure 3. As is evident in Figure 3B, there is a distinct atrial gender bias, with males exhibiting overall higher expression levels of almost all transcripts quantified as compared with females. In the ventricles, there is also a slight male bias in overall relative transcript expression levels (Figure 3C), although in contrast with the atria the pattern emerges in nonfailing hearts, as opposed to failing LV. Interestingly, previous studies in wild-type mouse hearts demonstrated a similar male gender bias in overall atrial gene expression [36].

### Gender Dependent Atrial Remodeling

**Figure 4:**
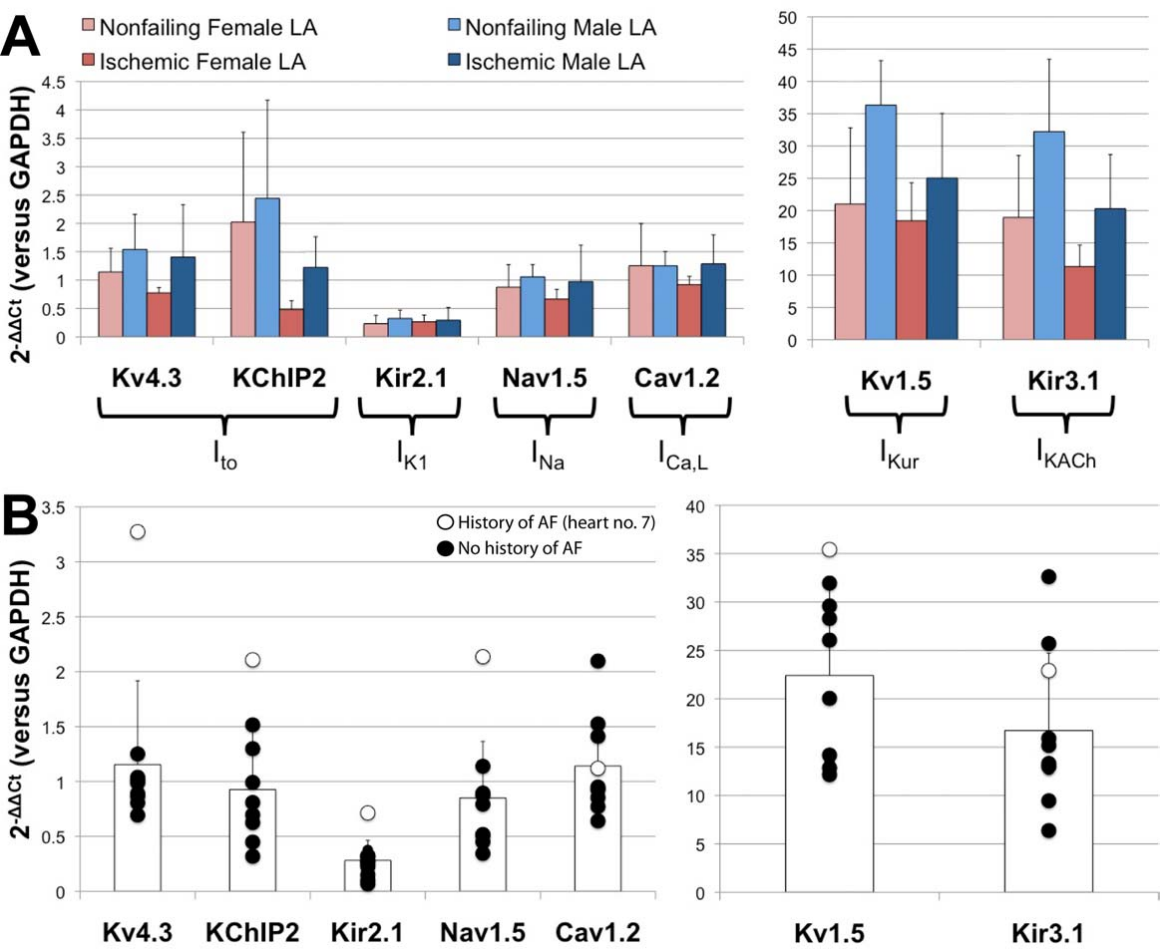
Gender dependent remodeling in the LA. (A) Gender dependent atrial remodeling of major ion channel subunits and accessory proteins in failing and nonfailing hearts of both genders. (B) Relative expression levels of failing LA samples of both genders. The white data points indicate the patient with a documented history of AF.

Because epidemiological data have revealed that males have an increased overall lifetime risk of developing atrial fibrillation [1], [2], we compared the expression levels of the major ion transcripts that have been shown or are postulated to undergo pathological remodeling [37], [38], [39]. The gender dependent relative expression levels of these transcripts are shown in Figure 4A.

Atrial fibrillation has been shown to be associated with significant reductions in I_to_ [40], I_Kur_ [41], I_KACh_ [42], and I_Ca,L_ [43], [44], whereas I_K1_ current tends to be increased in AF patients [42] and I_Na_ has been shown to not be significantly altered [45]. Surprisingly, when we assessed the gender dependence of these targets in our overall patient population, we observe patterns consistent with our female population being potentially more susceptible to the development of atrial fibrillation. As seen in Figure 4A, our female subjects exhibit lower expression levels in atrial K_v_4.3, KChIP2, K_v_1.5, and K_ir_3.1, as compared with our male subjects. We also see trends for lower expression in Na_v_1.5 and Ca_v_1.2, with no significant changes or trends in K_ir_2.1.

Based on the known clinical characteristics of our patient population, we identified one failing human heart (#7) with a documented history of AF from which we analyzed an LA specimen. Contrary to our expectations, as shown in Figure 4B, this particular sample had the highest relative expression levels of K_v_4.3, KChIP2, K_ir_2.1, Na_v_1.5, and K_v_1.5 when compared to all failing LA specimens of both genders. This samples does, however, have a lower relative expression level of Ca_v_1.2 than other male specimens in this group.

**Figure 5:**
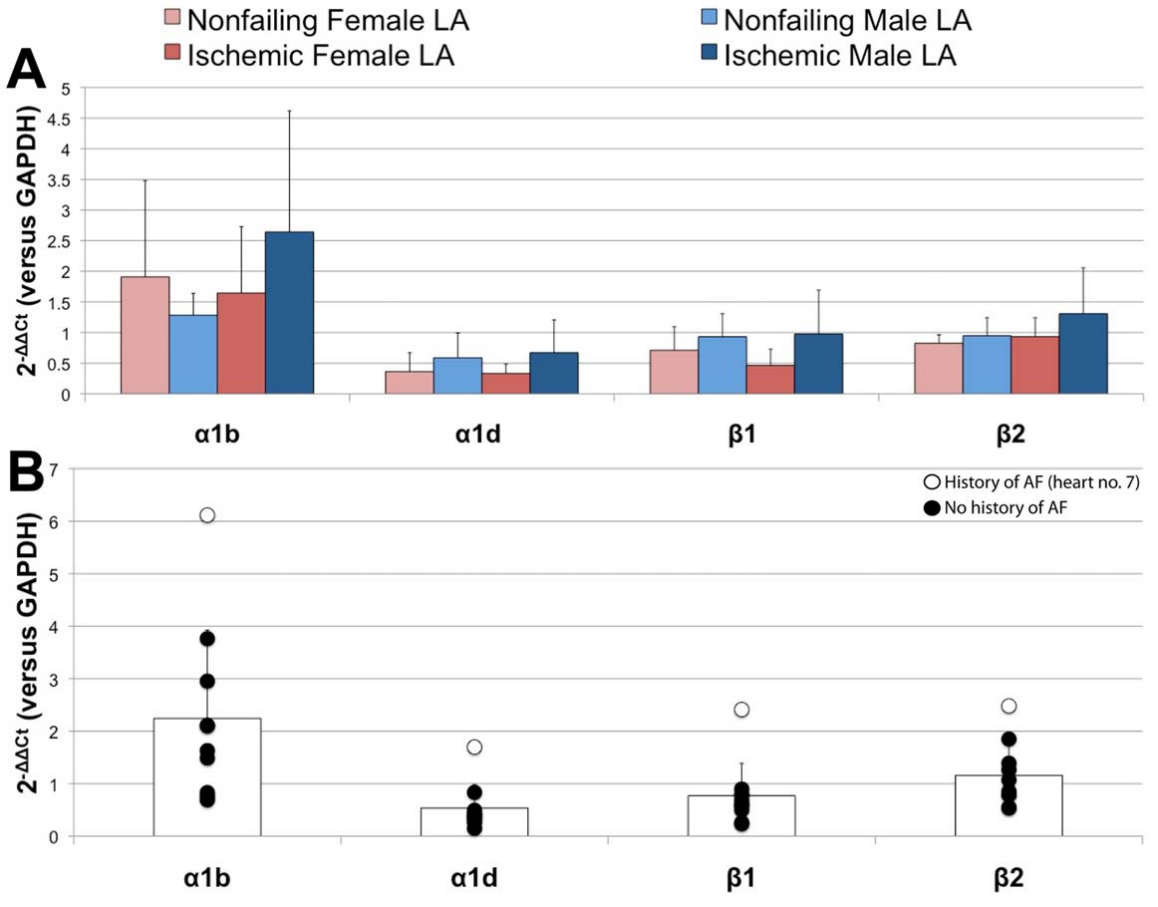
Gender dependent remodeling of adrenergic receptors. (A) Gender dependent atrial remodeling of adrenergic receptors in failing and nonfailing hearts of both genders. (B) Relative expression levels of adrenergic receptors in failing LA samples of both genders. The white data points indicate the patient with a documented history of AF.

We also explored the gender dependence of relative expression levels for adrenergic receptors, as an increased level of adrenergic activity can be associated with the onset of AF [46]. As seen in Figure 5A, we do not observe any significant differences in adrenergic receptor expression in the LA of failing and nonfailing hearts. There is an apparent trend that males tend to exhibit overall higher relative expression levels of α1b, α1d, β1, and β2 adrenergic receptors. This finding, however, may be due to an intrinsically decreased sympathetic tone in women as compared to men [47]. Interestingly, as shown in Figure 5B, the failing heart (#7) with a history of AF displays the highest relative expression levels of all of the adrenergic receptors analyzed.

### Gender Dependent Ventricular Remodeling

**Figure 6:**
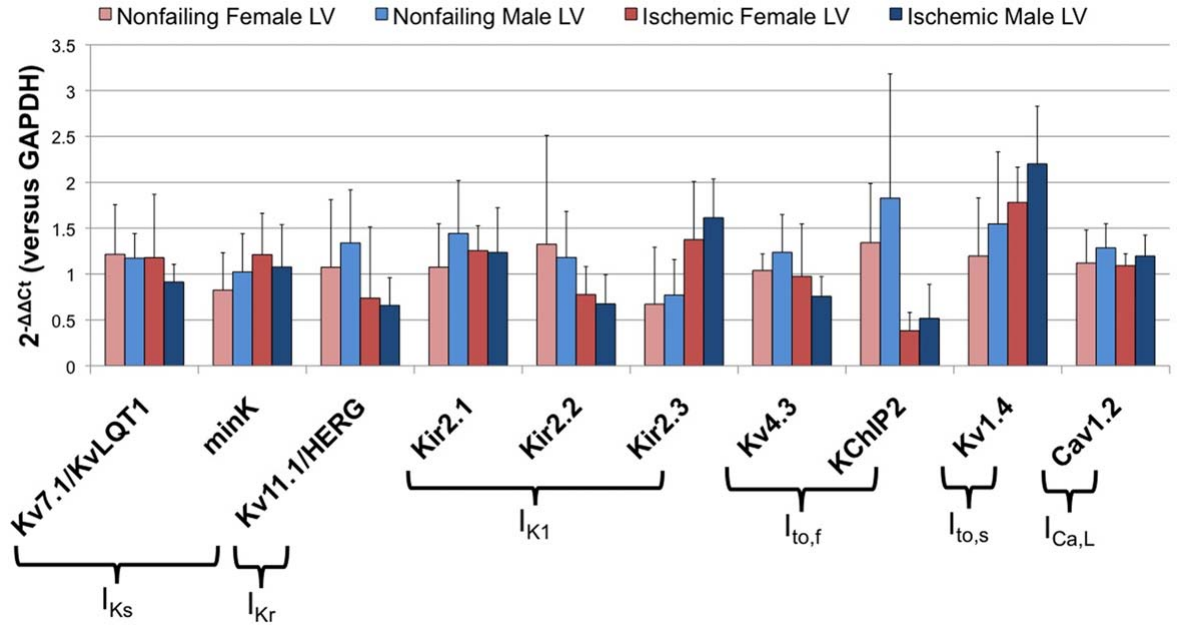
Gender dependent ventricular remodeling of major repolarizing ion channel subunits and accessory proteins in failing and nonfailing hearts of both genders.

**Figure 7:**
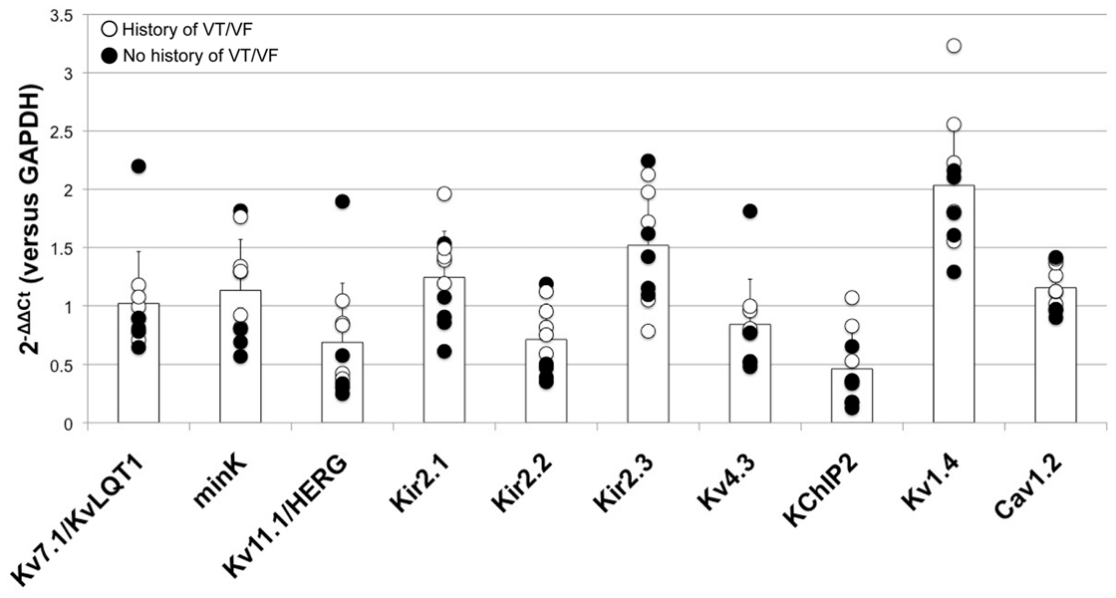
Relative expression levels of failing LV samples of both genders. The white data points indicate patients with a documented history of ventricular tachyarrhythmias.

Epidemiologically, females tend have a higher incidence of LQTS and drug-induced Torsades de Pointes [3], [4]. Subsequent analysis therefore focused on a subset of ion channels known to be important in cardiac repolarization [8], [48]. As shown in Figure 6, quantification of the relative expression levels of these transcripts revealed no significant differences in patterns based on gender. In addition, direct comparison of the expression levels of these transcripts linked to repolarization in LV samples from failing hearts with and without histories of ventricular tachyarrhythmias also do not reveal any striking patterns as shown in Figure 7.

### Gender Dependent Transmural Heterogeneity

**Figure 8:**
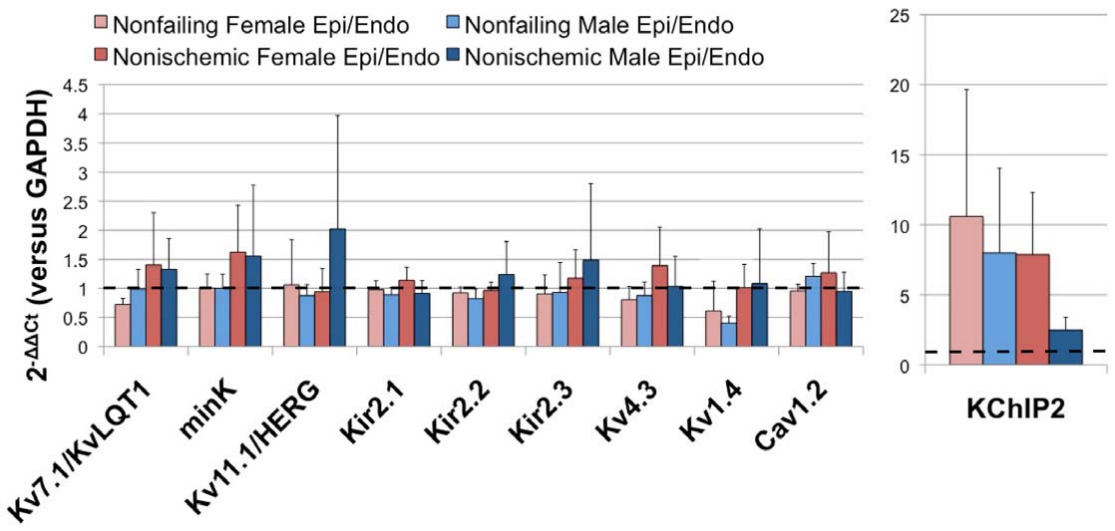
Average ratios of epicardial to endocardial expression of repolarization targets in failing and nonfailing hearts of both genders. The dotted back line represents a ratio of one.

Transmural heterogeneities in action potential waveforms across the ventricular wall are major determinants of arrhythmia susceptibility [49] and directly alter electrophysiological parameters during heart failure [23] Figure 8 shows the ratios of epicardial to endocardial expression for key transcripts linked to repolarization in failing and nonfailing hearts of both genders. Ratios of around one indicate those transcripts, such as K_v_7.1/K_v_LQT1, mink, K_ir_2.1, K_ir_2.2, K_ir_2.3, K_v_4.3, and Ca_v_1.2, that are uniformly expressed across the transmural wall of the LV in both failing and nonfailing hearts. On the other hand, ratios of either greater or less than one represent transcripts that are more heterogeneously expressed across the LV transmural wall. For example, KChIP2, the accessory protein for I_to_ channels, exhibits a significantly larger ratio than any of the other targets shown in Figure 8. In the failing male heart, however, this heterogeneity is not present as the ratio of epicardial to endocardial expression is one. Kv1.4, a pore-forming subunit for I_to,slow_, displays a reduced ratio in nonfailing hearts, but again, as with KChIP2, the transmural expression ratio is one in the failing heart.

## Discussion

In the present study, we explored the impact of gender on the transcript expression levels of the major cardiac ion channels, calcium handling proteins, and transcription factors in the failing and nonfailing human heart. We found gender-specific differences in relative expression levels of ion channel subunits, such as K_v_4.3, KChIP2, K_v_1.5, and K_ir_3.1 in the LA, but no significant gender-specific differences in relative expression levels of these subunits in the LV. Analyses of transmural heterogeneities in transcript expression levels in the LV, however, revealed a heterogeneity of expression across the LV wall in of the I_to,slow_ pore-forming subunit, K_v_1.4, as well as with the I_to,fast_ accessory subunit, KChIP2. In the nonfailing heart, K_v_1.4 exhibited higher expression in the epicardium of the nonfailing heart, whereas KChIP2 exhibited a stronger expression in the epicardium across gender and disease state.

Until recently, there has been very limited data available regarding global gender dependent cardiac ion channel gene expression in the human heart. A 2010 study by Gaborit, et al [18] explored gender dependent differences in the nonfailing human ventricle (left and right ventricles) and, unlike in our investigation, found significantly lower expression levels in the female LV of key ion channels and accessory proteins important in cardiac repolarization. Their patient population consisted of 10 men and 10 women with average ages of 38±12 years and 43±13 years, respectively. Our patient population, on the other hand, consisted of both failing and nonfailing human hearts with significantly older average ages (failing females, 54±9 years; failing males, 56±9 years; nonfailing females, 51±11 years, failing males, 50±18 years). The variability of qPCR data has shown to be significantly increased in aged animals [50] and humans [51], which in our case may mask any intrinsic gender dependent expression differences in the LV in our study.

The use of an older patient population may, however, be more representative with respect to the gender dependent analysis of LA targets important in the generation and maintenance of AF, as AF is relatively uncommon before the age of 60 and 10% of the overall population develops AF by 80 years of age [52]. In addition, 1 in 4 heart failure patients develop AF within five years of diagnosis [53], so it is likely that relevant atrial remodeling processes [54] are well underway in our patient population. Interestingly, although overall men are more likely to develop heart failure [55], women diagnosed with heart failure are approximately 1.5 times more likely to develop AF than their male counterparts [56]. Additionally, the overall higher atrial expression levels of almost all transcripts analyzed in this study (Figure 3B) in the failing male hearts, as compared to failing female hearts, may mask any intrinsic gender-based differences.

Interpretation of these results, however, with respect to both the LA and LV must be considered in light of the available clinical information, as shown in Tables 1 and 2. As described in the Results section, we identified one patient (failing heart no. 7) with a history of AF. This patient exhibited high expression levels of K_v_4.3, KChIP2, K_ir_2.1, Na_v_1.5, and K_v_1.5 when compared with all other failing LA specimens of both genders, as shown in Figure 4B. This sample did, however display a relatively lower expression level of Ca_v_1.2 as compared with other specimens in the group, which is likely related to the patient’s four-month history of permanent AF prior to transplantation. It should also be noted that, prior to diagnosis of AF, this patient had been previously diagnosed with ventricular arrhythmias which concurrently played a role in the progression of heart failure and overall arrhythmia history. As previously reported, in human AF the primarily physiological remodeling results from changes in I_Ca,L_ and significant reductions of current density have only been reported after 18 months of persistent AF [57]. In addition, as shown in Figure 7, we observed no remarkable expression patterns in key repolarization targets in the LV of failing hearts with and without a history of ventricular tachyarrhythmias, which may in part be due to the individual etiologies of heart failure, which must be elucidated further in future studies.

Prolongation of the cardiac action potential is a hallmark of heart failure [58]. Furthermore, heterogeneous distribution of repolarization transmurally across the ventricular wall manifests as electrophysiological heterogeneities which may also be pro-arrhythmic [49]. In our recent study, we provide direct experimental evidence of transmural heterogeneites in action potential waveforms in the human LV wedge preparation [23]. In the present study, as shown in Figure 8, we explored the potential molecular mechanisms of this functional heterogeneity. The data reveal expression gradients in KChIP2 expression across the transmural wall consistent with differences in I_to_ [49]. We were unable, however, to pinpoint other channels potentially contributing to action potential heterogeneity as the transcripts encoding the other ion channels prominent in repolarization were found to be more homogenously expressed across the LV wall. In addition, we did not find connexin 43 transcript expression to be heterogeneously expressed, although previous reports have demonstrated significantly lower epicardial connexin 43 protein expression in both failing and nonfailing hearts in our previous study [23]. Patterns of molecular heterogeneity directly affecting action potential durations may either be due to the presence of smaller “islands” of differential gene expression and function [23], [59] not captured in this study or post-translational protein modifications [60] not assessed in our transcript level investigation.

The wealth of information gathered from large-scale gene expression studies such as this and others [17], [19], not only provides insight into the gender dependent, pathological, or regional changes in ion channel expression, but also provides solid physiological data that can be incorporated into computational models. Furthermore, access to detailed clinical information, such as that presented this study, will both assist in interpretation of the molecular mechanisms involved in heart failure and arrhythmogenesis, as well as contribute to the development of tools for patient-specific computational models of cardiac electrophysiology. Constructing models of comprehensive human electromechanical activity requires a thorough understanding of the distribution of ion channels, calcium handling proteins, and other transporters in various regions of the heart, as well as their intrinsic modifications due to disease and gender as we have investigated in this study. Our colleagues Walmsley, et al in their paper “mRNA expression levels in failing human hearts predict cellular electrophysiological remodeling using a population-based simulation study” in this issue of the journal, present a first step towards patient-specific modeling of the action potential and calcium transient based on the comprehensive set of molecular data presented here.

### Study Limitations

The study of human hearts, while providing a unique opportunity to explore both the structural and functional mechanisms pertaining to arrhythmias, heart failure, and other pathological conditions, is limited by number of explanted hearts available. Nonfailing, donor hearts are especially precious and cannot be truly considered normal, control hearts due to that fact that they have been rejected for transplantation due to, in some cases, cardiac reasons. In addition, the underlying patient circumstances (i.e. medication, previous cardiac surgery, etc) cannot be controlled for. Thus, the hearts presented in this study represent the best available specimens.

### Conclusion

Epidemiologically, it is clear there is a gender disparity in arrhythmia incidence. To attempt to elucidate the underlying molecular mechanisms of these differences, we have explored the gender dependent expression of various ion channels, calcium handling proteins, and other transcription factors in the failing and nonfailing human heart in this study. While we observed significant gender dependent differences in atrial tissues, we did not however observe clear patterns of gender dependent expression in the ventricles. Our data do, however, emphasize the importance of addressing such confounding factors as gender, age, and disease progression when exploring ionic remodeling processes in the human heart and in constructing patient-specific models of cardiac electrophysiology.

## Supporting Information

Figure S1 **Hierarchical Cluster Analysis.** (A) Cluster analysis of ischemic and nonfailing LA/LV samples of both genders showing distinct separation based on cardiac location. (B) Cluster analysis of ischemic, nonischemic, and nonfailing LV samples showing no clustering. (C) Cluster analysis of nonischemic and nonfailing epicardial/endocardial samples also showing no clustering.
(TIF)

Table S1 **Custom-designed Taqman low-density gene array targets.**
(DOCX)
