# Supplementary figures and images for: Gender Differences in Electrophysiological Gene Expression in Failing and Non-Failing Human Hearts
Source: PLoS One. 2013 Jan 23;8(1):e54635. doi: 10.1371/journal.pone.0054635 (PMC3552854; doi:10.1371/journal.pone.0054635)

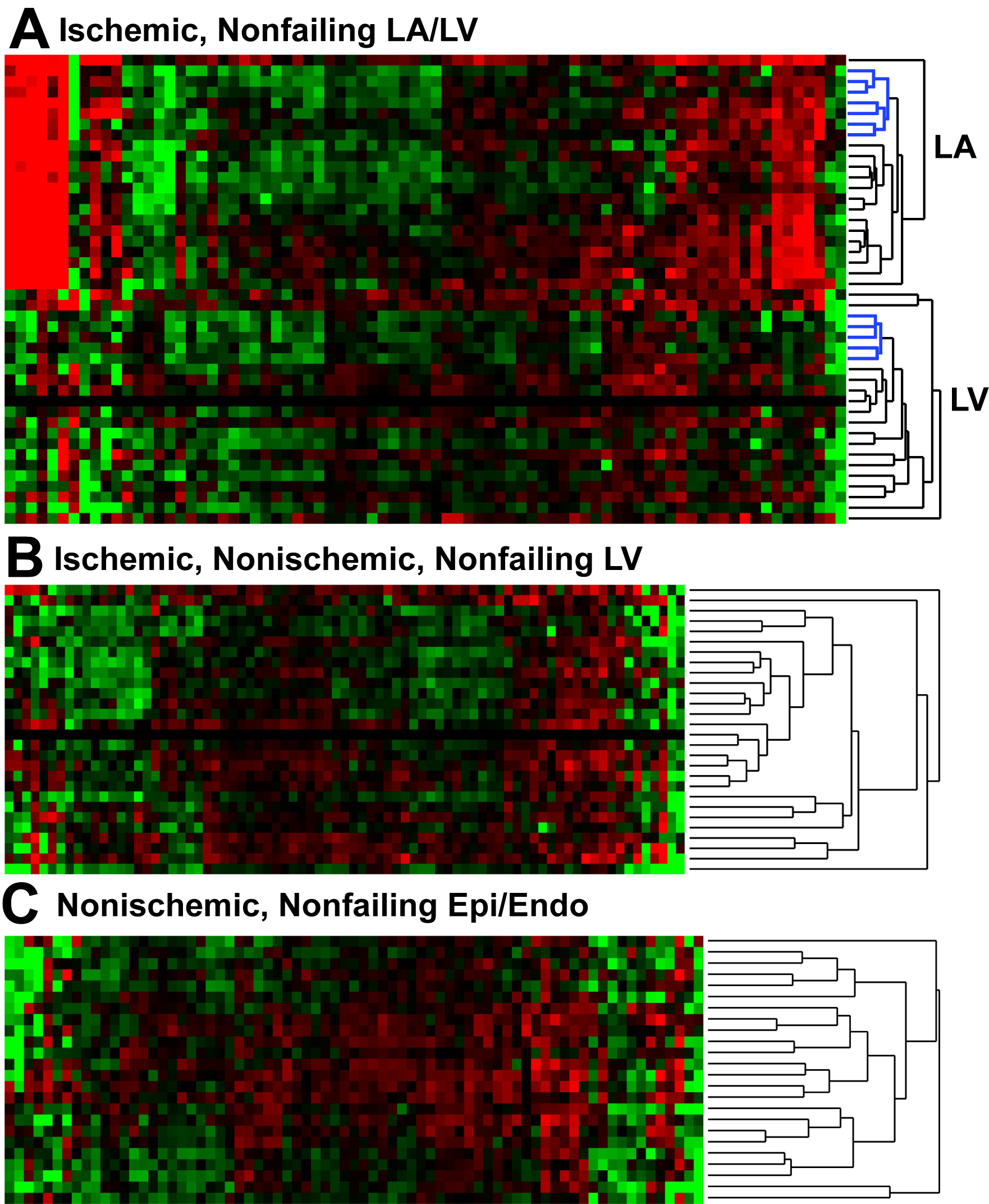

Supplement: Figure S1 — Hierarchical Cluster Analysis. (A) Cluster analysis of ischemic and nonfailing LA/LV samples of both genders showing distinct separation based on cardiac location. (B) Cluster analysis of ischemic, nonischemic, and nonfailing LV samples showing no clustering. (C) Cluster analysis of nonischemic and nonfailing epicardial/endocardial samples also showing no clustering. (TIF) [file pone.0054635.s001.tif]
