# Supplementary material for: Gender Differences in Electrophysiological Gene Expression in Failing and Non-Failing Human Hearts
Source: PLoS One. 2013 Jan 23;8(1):e54635. doi: 10.1371/journal.pone.0054635 (PMC3552854; doi:10.1371/journal.pone.0054635)
Supplement: Table S1 — Custom-designed Taqman low-density gene array targets. (DOCX) [file pone.0054635.s002.docx]

**Table S1. Custom-designed Taqman low-density gene array targets.**

| **Gene Name** | **Assay ID** |
| --- | --- |
| SUR1 (ABCC8) | Hs00165861_m1 |
| SUR2 (ABCC9) | Hs00245832_m1 |
| α-actinin (ACTN1) | Hs00241650_m1 |
| α1b Adrenergic Receptor (ADRA1B) | Hs00171263_m1 |
| α1d Adrenergic Receptor (ADRA1D) | Hs00169865_m1 |
| β1 Adrenergic Receptor (ADRB1) | Hs00265096_s1 |
| β2 Adrenergic Receptor (ADRB2) | Hs00240532_s1 |
| β3 Adrenergic Receptor (ADRB3) | Hs00609046_m1 |
| α1 subunit of Na/K Pump (ATP1A1) | Hs00167556_m1 |
| α2 subunit of Na/K Pump (ATP1A2) | Hs00265131_m1 |
| α3 subunit of Na/K Pump (ATP1A3) | Hs00265163_m1 |
| β1 subunit of Na/K Pump (ATP1B1) | Hs00426868_g1 |
| β2 subunit of Na/K Pump (ATP1B2) | Hs00155922_m1 |
| SERCA2a (ATP2A2) | Hs01566028_g1 |
| PMCA4 (ATP2B4) | Hs00608066_m1 |
| Ca_v_1.2 (CACNA1C) | Hs00167681_m1 |
| Ca_v_1.3 (CACNA1D) | Hs00167753_m1 |
| Ca_v_3.1 (CACNA1G) | Hs00367969_m1 |
| Ca_v_3.2 (CACNA1H) | Hs00234934_m1 |
| Calmodulin 3 (CALM3) | Hs00270914_m1 |
| CAM Kinase II Beta (CAMK2B) | Hs00365799_m1 |
| Calsequestrin 2 (CASQ2) | Hs00415779_m1 |
| Caveolin 3 (CAV3) | Hs00154292_m1 |
| CD4 | Hs00181217_m1 |
| Choline Acetyltransferase (CHAT) | Hs00252848_m1 |
| M1 Muscarinic Receptor (CHRM1) | Hs00912795_m1 |
| M2 Muscarinic Receptor (CHRM2) | Hs00265208_s1 |
| M3 Muscarinic Receptor (CHRM3) | Hs00327458_m1 |
| M4 Muscarinic Receptor (CHRM4) | Hs00265219_s1 |
| Calponin 1 (CNN1) | Hs00154543_m1 |
| GAPDH | Hs00266705_g1 |
| Connexin 43 (GJA1) | Hs00748445_s1 |
| Connexin 40 (GJA5) | Hs00270952_s1 |
| Connexin 45 (GJC1) | Hs00271416_s1 |
| HCN1 | Hs00395037_m1 |
| HCN2 | Hs00606903_m1 |
| HCN4 | Hs00175760_m1 |
| Iroquois 3 (IRX3) | Hs00735523_m1 |
| Inositol 1,4,5-Triphosphate Receptor 1 (ITPR1) | Hs00181881_m1 |
| Inositol 1,4,5-Triphosphate Receptor 3 (ITPR3) | Hs00609908_m1 |
| K_v_1.2 (KCNA2) | Hs00270656_s1 |
| K_v_1.4 (KCNA4) | Hs00357903_s1 |
| K_v_1.5 (KCNA5) | Hs00266898_s1 |
| K_v_1.6 (KCNA6) | Hs00266903_s1 |
| K_v_β1 (KCNAB1) | Hs00963155_m1 |
| K_v_β2 (KCNAB2) | Hs00186308_m1 |
| K_v_β3 (KCNAB3) | Hs00190986_m1 |
| K_v_2.1 (KCNB1) | Hs00270657_m1 |
| K_v_3.4 (KCNC4) | Hs00428198_m1 |
| K_v_4.2 (KCND2) | Hs00273378_m1 |
| K_v_4.3 (KCND3) | Hs00542597_m1 |
| mink (KCNE1) | Hs00264799_s1 |
| MIRP1 (KCNE2) | Hs00270822_s1 |
| MIRP2 (KCNE3) | Hs00538801_m1 |
| MIRP3 (KCNE4) | Hs00298953_m1 |
| K_v_11.1/HERG (KCNH2) | Hs00165120_m1 |
| KChIP2 (KCNIP2) | Hs01552688_g1 |
| K_ir_6.2 (KCNJ11) | Hs00265026_s1 |
| K_ir_2.2 (KCNJ12) | Hs00266926_s1 |
| K_ir_2.1 (KCNJ2) | Hs00265315_m1 |
| K_ir_3.1 (KCNJ3) | Hs00158421_m1 |
| K_ir_2.3 (KCNJ4) | Hs00705379_s1 |
| K_ir_3.4 (KCNJ5) | Hs00168476_m1 |
| K_ir_6.1 (KCNJ8) | Hs00270663_m1 |
| TWIK-1 (KCNK1) | Hs00158428_m1 |
| TREK-1 (KCNK2) | Hs00247951_m1 |
| TASK-1 (KCNK3) | Hs00605529_m1 |
| TWIK-2 (KCNK6) | Hs00191390_m1 |
| K_v_7.1/K_v_LQT1 (KCNQ1) | Hs00923522_m1 |
| Atrial Natriuretic Peptide (NPPA) | Hs00383231_m1 |
| Pannexin 1 (PANX1) | Hs00209791_m1 |
| Pannexin 2 (PANX2) | Hs00364525_m1 |
| KChAP (PIAS3) | Hs00180666_m1 |
| Phospholipase A2 (PLA2G4C) | Hs00234345_m1 |
| Phospholipase A2 (PLA2G6) | Hs00185926_m1 |
| Phospholamban (PLN) | Hs00160179_m1 |
| Protein Phosphatase 3 (PPP3CA) | Hs00174223_m1 |
| Protein Tyrosine Kinase 2 (PTK2B) | Hs00169444_m1 |
| Ryanodine Receptor 2 (RYR2) | Hs00181461_m1 |
| Ryanodine Receptor 3 (RYR3) | Hs00168821_m1 |
| Na_v_1.1 (SCN1A) | Hs00374696_m1 |
| Na_v_β1 (SCN1B) | Hs00962350_m1 |
| Na_v_β2 (SCN2B) | Hs00394952_m1 |
| Na_v_β3 (SCN3B) | Hs00393218_m1 |
| Na_v_1.5 (SCN5A) | Hs00165693_m1 |
| Na_v_2.1 (SCN7A) | Hs00161546_m1 |
| Na_v_1.7 (SCN9A) | Hs00161567_m1 |
| Sodium-Calcium Exchanger 1 (SLC8A1) | Hs00253432_m1 |
| Sarcolipin (SLN) | Hs00161903_m1 |
| T-box 2 (TBX2) | Hs00172983_m1 |
| T-box 20 (TBX20) | Hs00396596_m1 |
| T-box 3 (TBX3) | Hs00195612_m1 |
| T-box 5 (TBX5) | Hs01052563_m1 |
| Tyrosine Hydroxylase (TH) | Hs01002182_m1 |
| Vimentin (VIM) | Hs00185584_m1 |
